# Evidence Gap in Managing Lateral Pelvic Lymph Nodes in Rectal Cancer: a Systematic Review of Radiation Boost Strategies

**DOI:** 10.1007/s12029-026-01580-1

**Published:** 2026-09-07

**Authors:** Sedat Alp Pinar, Tin-Ning Wong, Timothy Mitchell, Chia Yew Kong

**Affiliations:** 1 Academic Unit of Surgery, Glasgow Royal Infirmary and University of Glasgow, 8-16 Alexandra Parade, Glasgow, G31 2ER United Kingdom; 2 Department of Oncology, The Beatson West of Scotland Cancer Centre, Glasgow, United Kingdom

**Keywords:** Rectal cancer, Pelvic lymph nodes, Radiotherapy boost, Dose escalation, Simultaneous-intensity boost, Radiation oncology

## Abstract

**Purpose:**

Lateral pelvic lymph node (LPLN) involvement is a significant predictor of local recurrence in patients with locally advanced rectal cancer (LARC). While lateral pelvic lymph node dissection (LPLND) is routinely used in some countries to manage suspicious nodes, it is associated with increased morbidity and is not widely adopted in Western practice. Radiation boost (dose escalation) to involved LPLNs during neoadjuvant chemoradiotherapy (nCRT) has emerged as a potential non-surgical alternative. Despite increasing adoption of radiation boost to clinically involved LPLNs, there remains limited evidence defining its safety, oncologic benefit, and role relative to LPLND.

**Methods:**

A systematic search of MEDLINE, EMBASE, ClinicalTrials.gov, and Cochrane databases was conducted following PRISMA guidelines. Studies were included if they reported outcomes of radiation dose escalation specifically targeting radiologically suspicious LPLNs in the context of nCRT.

**Results:**

Ten retrospective cohort studies encompassing 482 radiation boosted patients were included. Boost doses ranged from 35.0 to 60.2 Gy. Rates of Grade 2–3 toxicity ranged from 28.0% to 39.3% across individual studies, with only one study reporting a single Grade 4 adverse event. Across individual studies, reported nodal response rates ranged from 62.3% to 100%. Comparative studies suggest that radiation boost may improve local control and reduce LPLN recurrence.

**Conclusion:**

Current retrospective evidence suggests that radiation dose escalation to involved LPLNs is a promising treatment strategy; however, the available data are limited by retrospective study designs and substantial clinical heterogeneity. Given the absence of prospective evidence and lack of consensus in current guidelines, an important evidence gap remains. Well-designed prospective trials are warranted to define the role of LPLN boost relative to LPLND.

**Supplementary Information:**

The online version contains supplementary material available at https://doi.org/10.1007/s12029-026-01580-1.

## Introduction

**Fig. 1:**
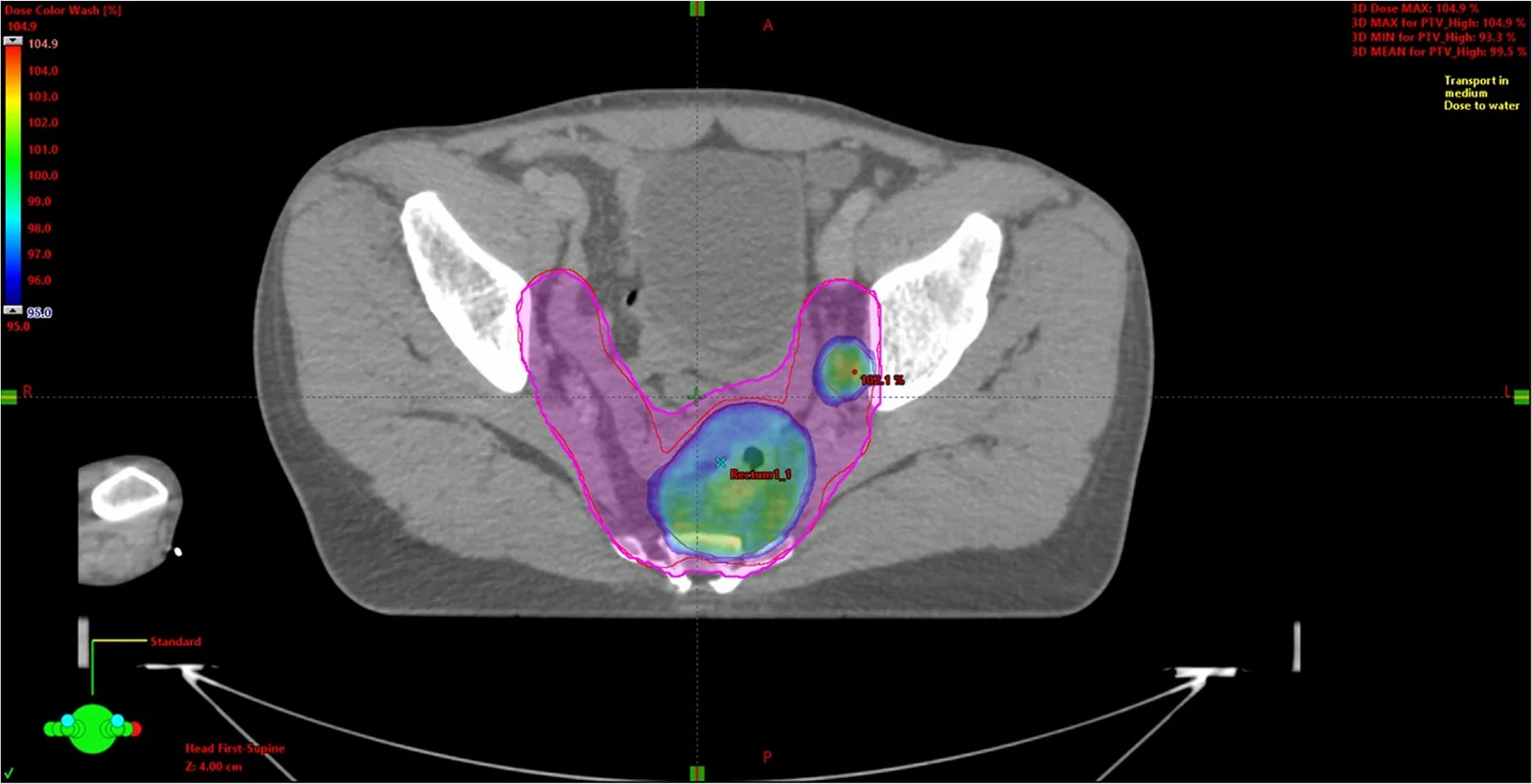
Radiotherapy planning MRI demonstrating standard pelvic irradiation fields and simultaneous integrated boost (SIB) regions. The purple region represents the standard elective pelvic radiotherapy volume, while the green region represents the selectively dose-escalated boost volume. The boosted volumes include the primary rectal tumour and a radiologically suspicious obturator lymph node within the lateral pelvic compartment. This illustrates the anatomical relationship between conventional pelvic fields and nodal boost volumes in contemporary clinical practice

In patients with locally advanced rectal cancer (LARC), the lateral pelvic sidewall is a frequent site of locoregional recurrence due to involvement of lateral pelvic lymph nodes (LPLNs) [1]. Persistently enlarged LPLNs following neoadjuvant therapy are associated with a substantial risk of lateral pelvic recurrence, with nodes measuring ≥ 7 mm demonstrating a reported recurrence risk of 15%, highlighting the need for effective strategies to improve nodal control [2]. Management strategies for these LPLNs, particularly those involving the internal iliac and obturator nodal stations, remain controversial and differ significantly between Asian centres and centres in Europe and North America [3]. In many parts of Asia, pelvic node involvement is accounted for by combining total mesorectal excision (TME) with the dissection of the pelvic lymph nodes (LPLND) prophylactically [3]. In contrast, Western centres employ a neoadjuvant chemoradiotherapy (nCRT) regimen followed by TME [3]. In the West, LPLND is reserved for cases with radiological evidence of nodal involvement, usually defined as lymph nodes with a short axis diameter of 7 mm or greater [4]. Emerging evidence also suggests a potential role for morphological features to predict node involvement such as internal heterogeneity, loss of fatty hilum, round shape, and irregular margins [5]. However, previous studies also showed that selecting patients for LPLND based on nodal size and morphological features may be inaccurate [6]. Furthermore, LPLND is associated with significant morbidity and a longer hospital stay than a TME-alone approach, which makes it imperative that patients are selected appropriately for the procedure [7]. In current neoadjuvant practice, these lateral pelvic nodes typically lie within the standard pelvic radiation field but are often outside the simultaneous integrated boost volume fields encompassing only the primary tumour and involved mesorectal nodes. Figure 1 illustrates this concept, showing our institutional approach where a radiologically suspicious LPLN is selectively targeted with an additional simultaneous integrated boost (SIB). This visual highlights the anatomical rationale for dose escalation and the potential opportunity to improve local control without surgical dissection.

Issues of imperfect prediction of node involvement magnifies the clinical need for effective diagnostic and therapeutic strategies for the management of LPLNs. In this review, we evaluate the potential role of delivering a radiation (RT) boost to these LPLNs in addition to conventional nCRT, aiming to offer a non-surgical alternative to managing suspected LPLNs.

These nodes lie within the elective pelvic radiation field and are treated with a standard 45.0 Gy dose in 25 fractions, in line with the current guidelines of the Royal College of Radiologists [8]. The guidelines also recommend the use of dose escalation of the intraluminal tumour with simultaneous integrated boost (SIB) to a dose of 50.0 Gy in LARC, however there is no guideline concerning the LPLNs if there is suspicion of involvement [8]. A similar gap is reflected in current ASTRO guidelines, which provide no specific recommendations regarding radiation dose escalation to lateral pelvic lymph nodes. While techniques such as simultaneous integrated boost (SIB) or sequential boost are mentioned in the context of the primary tumour and involved mesorectal nodes, their use in the lateral pelvic compartment is not addressed [9]. Furthermore, current ESTRO recommendations do not define specific criteria for LPLN boost selection, target delineation margins, or boost dose and fractionation regimens for involved lateral pelvic lymph nodes [10]. Despite the absence of clear guidance from these major organisations, several specialist centres, including ours, have adopted the practice of selectively delivering RT boost to radiologically suspicious LPLNs based on multidisciplinary team (MDT) consensus. This variation in practice underscores the existing evidence gap and the need for systematic evaluation of the safety and effectiveness of this approach.

Given that nCRT is already used to substitute prophylactic LPLND with comparable oncological outcomes, it may be plausible to use radiation dose escalation to treat involved LPLNs as an alternative to surgical node dissection. This systematic review aims to evaluate the current evidence on the use of RT boost to LPLNs in patients with LARC undergoing neoadjuvant chemoradiotherapy.

## Methods

**Fig. 2:**
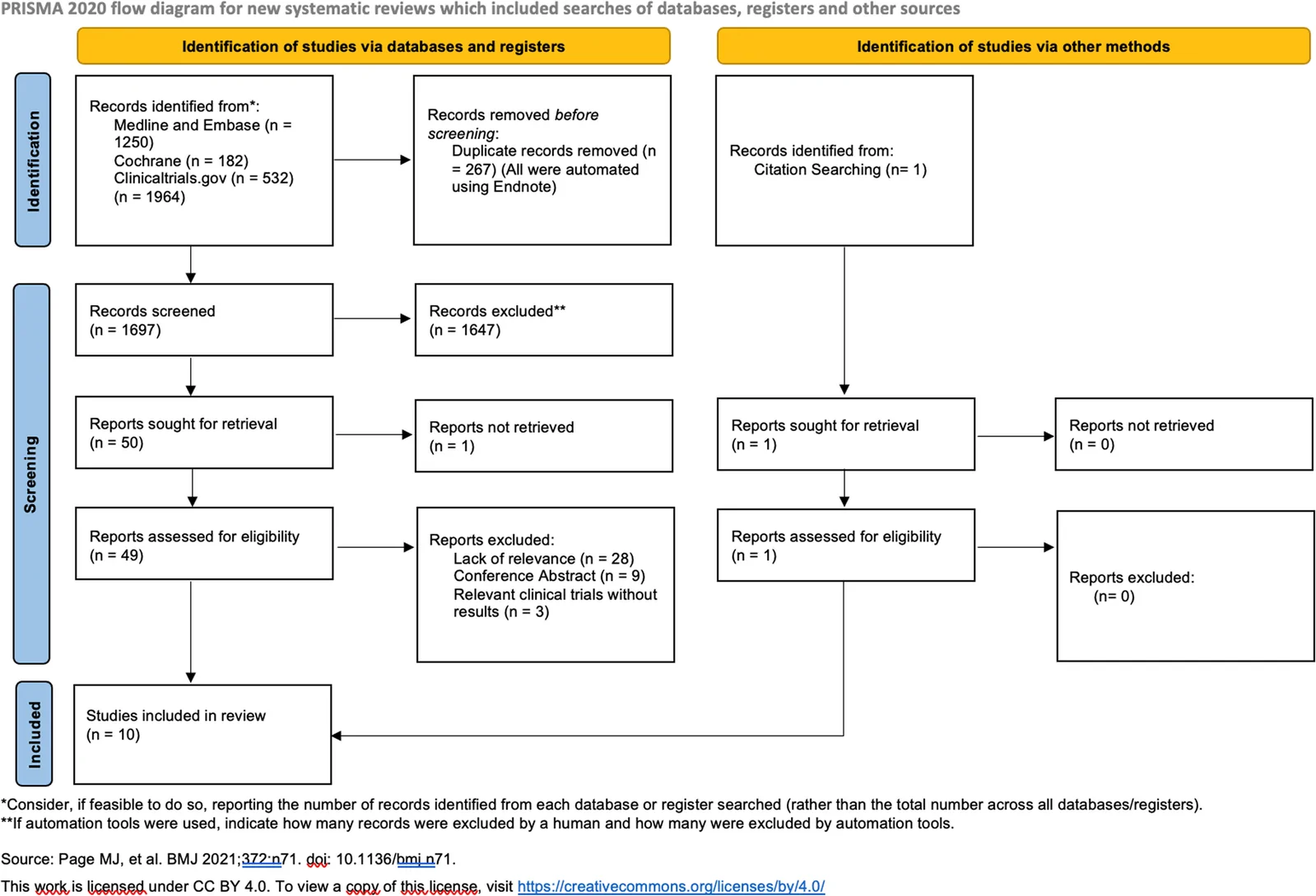
PRISMA flow diagram of the literature search and inclusion process

This systematic review was conducted in accordance with the PRISMA guidelines (Fig. 2) [11]. The review protocol was registered with PROSPERO (NIHR CRD420251087553) [12].

A literature search was conducted in July 2025 using MEDLINE (Ovid), EMBASE (Ovid), Clinicaltrials.gov, and Cochrane Reviews database. Search results were limited to the English language. References were managed using EndNote software (version 21.5; Clarivate Analytics, Philadelphia, PA, USA) [13].

Duplicates were removed and this was followed by the screening of retrieved titles and abstracts for eligibility.

The search strategies for each of the different databases used are presented in Supplement Table 1.

Studies were screened sequentially and independently by two co-authors, following PRISMA guidelines. The screening process is summarised in Fig. 2. Studies were deemed eligible if they reported on the treatment of LPLNs using any form of RT boost, including SIB or sequential boost strategies, and provided extractable data on at least one of the following outcomes: toxicity, dosimetry, nodal response, survival, or recurrence.

Studies were excluded if they were not available in English, lacked extractable data, or were published only as conference abstracts. Following eligibility assessment, all potentially relevant full-text articles were retrieved for detailed review. Reference lists of included studies were also manually searched to identify additional eligible papers.

Data extraction was performed independently by two reviewers using pre-piloted extraction forms. Any discrepancies were resolved through discussion and consensus to ensure accuracy and reproducibility. Extracted data included study characteristics (author, year, country, design, sample size), patient population, details of the RT boost technique, comparator (if applicable), and reported outcomes. Key oncological endpoints included overall survival (OS), disease-free survival (DFS), progression-free survival (PFS), metastasis-free survival (MFS), locoregional control, and LPLN response. Data on dosimetry and treatment-related toxicity were also systematically collected.

Given the heterogeneity of study designs and outcome measures, a narrative synthesis approach was adopted to summarise the findings, with a focus on feasibility, safety, and oncological efficacy of LPLN dose escalation.

Methodological quality and risk of bias were assessed independently by two reviewers, with any discrepancies resolved through consensus. Due to the inclusion of heterogeneous study designs, two distinct validated instruments were utilized. The Newcastle-Ottawa Scale (NOS) was used to evaluate comparative studies across three domains: selection (up to 4 points), comparability (up to 2 points), and outcome (up to 3 points), with a maximum attainable score of 9 points (Table 1) [14]. Conversely, the Methodological Index for Non-Randomized Studies (MINORS) checklist, restricted to the 8 non-comparative items (maximum attainable score of 16 points), was used to evaluate single-arm studies (Table 2) [15].

## Results

Our literature search identified one ongoing randomised controlled trial investigating the role of radiotherapy boost to radiologically involved LPLNs using SIB techniques [16, 17]. Study outcomes for this trial include pathological complete response (pCR) rates, surgical complexity, and toxicity. 

Among the eligible studies included in our review, all were retrospective cohort studies. NOS and MINORS scores for these studies are presented in Tables 1 and 2 respectively. Three comparative studies achieved the maximum Newcastle–Ottawa Scale (NOS) score of 9/9, while two studies scored 8/9. According to the Agency for Healthcare Research and Quality standards, these scores indicate good methodological quality. The non-comparative retrospective studies assessed using the MINORS instrument scored between 11 and 12 out of a maximum of 16, indicating generally good methodological quality. However, none of the studies achieved the maximum MINORS score, primarily because all studies were retrospective and no study reported a prospective sample size calculation to ensure adequate statistical power. All studies included in this review are summarized in Table 3. 

**Table 1:** Newcastle–Ottawa Scale (NOS) assessment of included cohort studies. The NOS was used to assess methodological quality across three domains: Selection (maximum 4 points), Comparability (maximum 2 points), and Outcome (maximum 3 points)

Study Title	Selection (4)				Comparability (2)	Outcome (3)			Raw NOS Score	Converted Scores to AHRQ Standards (Good, fair, poor).
	Representativeness of exposed cohort (1)	Selection of non-exposed cohort (1)	Ascertainment of exposure (1)	Demonstration that outcome of interest was not present at the start of the study (1)		Assessment of outcome (1)	Duration of follow-up (1)	Adequacy of follow-up cohort (1)		
Chen et al. [22]	1	1	1	1	2	1	0	1	8	Good
Hassanzadeh et al. [23]	1	1	1	1	2	1	1	1	9	Good
Li Zhang Yu et al. [18]	1	1	1	1	2	1	1	0	8	Good
Meldolesi et al. [26]	1	1	1	1	2	1	1	1	9	Good
Pang et al. [19]	1	1	1	1	2	1	1	1	9	Good

**Table 2:** Methodological quality assessment of non-comparative retrospective cohort studies using the Methodological Index for Non-Randomized Studies (MINORS). Each study was evaluated according to the eight items applicable to non-comparative studies; items were scored as 0 (not reported), 1 (reported but inadequate), or 2 (reported and adequate), with a maximum possible score of 16

Study Title	Clearly Stated Aim (2)	Inclusion (2)	Prospective Collection of Data (2)	Appropriate Endpoints (2)	Unbiased Assessment (2)	Appropriate Follow-up Period (2)	Follow-Up (2)	Prospective Calculation of Study Size (2)	Total Score
Büttner et al. [27]	2	2	0	2	2	2	2	0	12
Geng et al. [20]	2	2	0	2	2	2	2	0	12
Hartvigson et al. [24]	1	2	0	2	2	2	2	0	11
Li, Geng, Wang et al. [21]	2	2	0	2	1	2	2	0	11
Nayak et al. [25]	2	2	0	2	2	2	2	0	12

**Table 3:** Summary of Studies Reporting Outcomes Following Lateral Pelvic Lymph Node Radiation Boost. DR= Disease Recurrence; RT = Radiotherapy; SIB = Simultaneous Integrated Boost; Gy = Gray; DFS = Disease-Free Survival; OS = Overall Survival; LC = Local Control; LRR = Locoregional Recurrence; LRC: Loco-regional Control LLR = Lateral Local Recurrence; MFS = Metastasis-Free Survival; CTCAE = Common Terminology Criteria for Adverse Events; RTOG = Radiation Therapy Oncology Group; IMRT = Intensity-Modulated Radiotherapy; PTV = Planning Target Volume, GTVn= nodal gross tumour volume, CTV= clinical target volume

Author (Year)	Country	Study Design	*n* (boosted)	*n* (non-boosted)	RT Boost Dose/Technique	Control Treatment	PTV Margins	Outcomes Measured	Key Findings
Büttner et al [27]	Germany	Retrospective	27	n/a	Median dose of 60.2 Gy/ SIB	n/a	n/a	DFS; LC; OS; toxicity (CTCAE)	- Grade 3 or 4 toxicity related to SIB: 0% - 2-year OS: 80% - 2-year DFS: 80% - LC: 100%
Chen et al. [22]	United States of America	Retrospective	12	41 patients without clinical LPLN involvement	54.0–59.4 Gy/ SIB	External beam radiation therapy (45 Gy in 25 fractions) to the primary tumour and regional draining lymph node basins followed by a boost (5.4 Gy in 3 fractions) to gross disease	10–15 mm volume expansion	LPLN recurrence; LPLN response; OS; PFS; toxicity (CTCAE v5.0)	- 3- year OS: 90.04% vs. 83.33% (*p* = 0.890) in boosted vs. non-boosted respectively - 3-year PFS 80.12% vs. 80.21% (*p* = 0.529) in boosted vs. non-boosted respectively - LPLN recurrence: 0% in both groups - Acute Grade 2 + toxicity (75.0% vs. 41.5%) (*p* = 0.041) in boosted vs. non-boosted respectively - No difference in acute Grade 3 + toxicity
Geng et al. [20]	China	Retrospective	52	n/a	56.0–60.0 Gy/ SIB	n/a	5 mm margin to GTVp/GTVn and CTV	DFS; LRR; toxicity (CTCAE v4.0)	- 1-year DFS: 89.9% - 2-year DFS: 74.6% - Grade 2 or 3 radiation related toxicity: 28.8% - Grade 4 toxicity: 0% - 1-year LRR: 0% - 2-year LRR: 2.4%
Hartvigson et al. [24]	United States of America	Retrospective	12	n/a	53.48–60.2 Gy/ SIB or sequential boost	n/a	5–7 mm margin to GTVn and CTV	LC; OS; toxicity	- Acute grade 3 toxicity related to boost: 8.33% (perineal dermatitis and anaemia) - Grade 4 toxicity: 0% - Total acute Grade 2–3 toxicity related to boost: 25.0% - 1- year OS: 91.7% - 1- year LC: 90% - OS: 75.0% - Small bowel, bladder and femoral head doses were acceptable
Hassanzadeh et al. [23]	United States of America	Retrospective	30	8 patients with clinical LPLN involvement and 83 patients without clinical LPLN involvement	35.0 Gy/ SIB	SCRT to the whole pelvis with a dose of 5 Gy in 5 fractions	n/a	DR; regional recurrence; toxicity (CTCAE v5.0)	- Regional recurrence rates of 7% vs. 25% in boosted vs. non-boosted LPLN involved groups respectively - DR rates of 13% vs. 25% in boosted vs. non-boosted LPLN involved groups respectively - Acute grade 3 + GI toxicity: 3.33% - Late Grade 3 + GI toxicity: 10%
Li, Geng, Wang et al. [21]	China	Retrospective	75 (non-operative post-treatment MRI available in 23)	n/a	56.0–60.0 Gy/ SIB	n/a	5 mm margin to GTVp/GTVn and CTV	LPLN response; PFS; toxicity (CTCAE v4.0)	- 2-year PFS: 85.6% vs. 74.6% for non-operative vs. operative groups respectively - Acute grade 2/3 toxicity: 28% - Acute grade 4 toxicity: 0% - Nodal response (shrinkage from ≥ 7 mm to ≤ 5 mm): 100%
Li, Zhang, Yu et al. [18]	China	Retrospective	83	68 patients with clinical LPLN involvement	56.0–60.0 Gy/ SIB	IMRT- 50.6 and 41.8 Gy to PGTVp and PTV respectively	n/a	DFS; LPLN recurrence; post-operative complication rates; toxicity (CTCAE v4.0)	- Gade 2 radiation induced toxicity: 30.1% vs. 39.1% (*p* = 0.217) in the boosted vs. non boosted group respectively - Post-operative complication rates: 21.8% vs. 12.2% (*p* = 0.198) in the boosted vs. non boosted group respectively - 2-year LPLN recurrence rates: 0% vs. 10.8% (*p* = 0.024) in boosted vs. non-boosted groups respectively
Meldolesi et al. [26]	Italy	Retrospective	82	94 patients with clinical LPLN involvement	45.0 Gy/ SIB	71 patients received 55 Gy to primary tumour and 45 Gy to elective nodes; 23 patients received 25 Gy to both primary tumour and elective nodes	n/a	DFS, LC, MFS, OS	- 3 year OS: 96.0% vs. 85.0%; 5- year OS 90.0% vs. 75.0% (*p* = 0.006) - 3-year DFS 91% vs. 73.0%; 5-year DFS 88.0% vs. 68.0% (*p* = 0.03) - 3-year LC was 99.0% vs. 88.0%; 5-year LC was 97.0% vs. 86.0% (*p* = 0.026)
Nayak et al. [25]	India	Retrospective	61	n/a	Median dose of 57.0 Gy/ SIB	n/a	5 mm around CTV, and 5–7 mm around GTVn.	LPLN response; OS; MFS; toxicity (RTOG)	- Nodal response: 62.3% - 3-year DMFS: 84.4% - 3-year OS: 95.1% - Grade 2 toxicity: 31.1% - Grade 3 toxicity: 8.2% - Grade 4 toxicity: 0%
Pang et al. [19]	China	Retrospective	48	154 patients with clinical LPLN involvement (60 underwent nCRT and 94 nCT)	Median dose of 58.0 Gy/ SIB	Doses of 56–58, 50, and 45 Gy were delivered to PTVGTVnd, PTV-GTV, and PTV-CTV at 25 fractions, respectively	8 mm margin around the GTV, GTVn, and CTV	LPLN response; LLR; LR; toxicity (CTCAE v4.0)	- Nodal response rate: 72.9% vs. 65.0% vs. 48.9% in the boosted, nCRT and nCT groups respectively (*p* = 0.013). - Grade 3 and 4 toxicity: 29.1% vs. 16.6% in the SIB vs. nCRT groups (*p* = 0.121). - 3- year LLR: 2.3% vs. 20.4% vs. 31.6% in the boosted, nCRT and nCT groups respectively (*p* < 0.001)

### Study Characteristics

Our review included studies from 5 different countries. 40% (*n* = 4) of the studies included were from China [18–21], with 30% (*n* = 3) being from the USA [22–24].

Across all studies, 482 LARC patients received RT boost to the LPLNs; with the largest contribution coming from Li et al. [18] with 83 patients.

Total treatment dose with boost ranged from 35.0 Gy to 60.2 Gy. All studies used SIB, although some patients received a sequential boost instead [24]. Only six studies reported PTV margins (planning target volume), with these ranging from 5 mm to 15 mm [19–22, 24, 25].

Toxicity was the most reported outcome measure, with 90% (*n* = 9) of studies reporting Common Terminology Criteria for Adverse Events (CTCAE) or Radiation Therapy Oncology Group (RTOG) graded toxicity outcomes. OS in relation to RT-boost was reported by 50% (*n* = 5) of the studies [22, 24–27], whereas 40% (*n* = 4) reported outcomes on DFS [18, 20, 26, 27], and 30% (*n* = 3) reported LPLN response rates [19, 21, 25].

### LPLN Response, Local Control and Local Recurrence

Three studies (30%) reported their LPLN response outcomes following treatment with RT boost, with differing results [19, 21, 25]. Li et al. [21], although not defining nodal response, reported a nodal shrinkage rate of 100% in a cohort of 23 non-operative patients with LPLNs ≥ 7 mm. Using a concrete definition, categorising “good responders” as those whose nodes reduced to < 5 mm following nCRT, Nayak et al. [25] reported a response rate of 62.3%. The two studies had different definitions of “suspicious” nodes in the lateral pelvic compartment, with a size threshold of ≥ 7 mm for Li et al. [21] and ≥ 5 mm in Nayak et al. [25], with varying morphological features. Pang et al. [19], again defining response as size shrinkage to < 5 mm, compared nodal response in SIB vs. non-SIB patients, finding that the cohort with nodal boost has a greater response rate of 72.9% vs. 48.9% respectively (*p* = 0.007).

Three studies reported outcomes related to local control. Büttner et al. [27] reported a local control rate of 100%, defined as the absence of progression of the dose-escalated lesion on imaging; however, the duration of follow-up for this outcome was not specified. Hartvigson et al. [24] reported a 1-year local control rate of 90% using a similar definition. Meldolesi et al. [26] provided comparative data between patients receiving RT boost versus those who did not. The 3- and 5-year local control rates were 99% vs. 88% and 97% vs. 86%, respectively, in favour of the RT boost group (*p* = 0.026) [26].

In relation to local control, several studies reported local and LPLN recurrence outcomes. In the cohort of Geng et al. [20], there were no local recurrences at 1 year, and the rate of local recurrence was only 2.4% at 2- years, with there being no reported recurrences in the pelvic nodal drainage area that received RT boost. Similarly, lateral local recurrence rates were higher in the non-SIB nCRT and non-SIB nCT cohorts reported by Pang et al., [19] with 20.4% and 31.6% compared to just 2.3% in the SIB treated group respectively (*p* < 0.001).

Li et al. [18] showed direct benefit of RT boosting the nodes in their study, with 2- year LPLN regrowth rates of 0% vs. 10.8% in the SIB and non-SIB treated groups respectively (*p* = 0.024). Chen et al. [22] reported no LPLN recurrences in 3-years following treatment. Hassanzadeh et al. [23] also reported lower regional recurrence rates for the RT boosted cohort (7% vs. 25%).

### Survival

Two studies compared OS in the SIB-treated vs. non-SIB-treated cohorts. Chen et al. [22] found a 3- year OS rate of 90.04% vs. 83.33% (*p* = 0.890); this was reported to be 96% vs. 85% (*p* = 0.006) in the cohort of Meldolesi et al. [26] in the SIB-treated vs. non-SIB-treated cohorts respectively. Hartvigson et al. [24] reported a 1- year OS rate of 90% in their cohort [24]. Geng et al. reported DFS rates of 89.9% and 74.6% respectively at 1- and 2- years. Meldolesi et al. [26] compared 3- and 5- year DFS rates in their RT-boosted vs. non-boosted cohorts, with the RT boosted group performing significantly better (91% vs. 73% at 3- years; 88% vs. 68% at 5- years (*p* = 0.03)). The same study also reported superior MFS outcomes for the RT boosted group at 3- and 5- years (91% vs. 82%; 91% vs. 75% (*p* = 0.04)) [26].

### Toxicity

Although nine studies (90%) reported toxicity outcomes, seven (70%) used Common Terminology Criteria for Adverse Events (CTCAE) grading. However, due to heterogeneity in reporting methods, including inconsistencies in distinguishing between SIB-related and overall treatment toxicity, as well as the lack of separation between acute and late events in some studies, pooled analysis of toxicity data was not feasible. Nonetheless, individual study findings provide valuable insights into the safety profile of LPLN dose escalation and are summarised below.

Büttner et al. [27] showed that none of the Grade 3 toxicities observed were attributable to the RT boost. Hartvigson et al. [24] documented only one Grade 3 event (perineal dermatitis and anaemia) which was directly linked to RT boost. Nayak et al. [25] observed that the most frequently faced toxicities related to radiation boost were moist desquamation and enterocolitis, each with an incidence of 4.3%.

Pang et al. [19] provided the comparative evidence in the difference of Grade 3/4 toxicities between patients undergoing RT boost vs. those that underwent conventional nCRT, showing that the boosted cohort had a higher rate of events with 29.1% vs. 16.6%, albeit statistically insignificant (*p* = 0.121). Similarly, Chen et al. [22] showed no significant difference in the rates of CTCAE graded toxicity rates between SIB-treated and non-SIB-treated patients.

Hassanzadeh et al. [23], although not reporting rates of acute graded toxicity events specifically in the RT-boosted cohort, reported a rate of 10% chronic Grade 3 and 3.33% Grade 4 toxicity events, the latter being a case of small bowel obstruction.

Reporting of organ-at-risk (OAR) constraints was inconsistent across studies. Where described, OAR considerations included dose exposure to the small bowel, sigmoid colon, bladder, femoral heads, and pelvic soft tissues. Hassanzadeh et al. specified planning objectives including a small bowel maximum dose < 25 Gy, bladder maximum dose < 38 Gy, bladder V20 Gy < 15 mL, and femoral head V30 Gy < 10 mL, with small bowel constraints considered mandatory [23]. Pang et al. reported specific planning constraints including bowel bag V50 ≤ 5%, bladder V50 ≤ 50%, and femoral heads V50 ≤ 5%, while Geng et al. reported that OAR constraints were based on the QUANTEC recommendations [19, 20, 28]. Hartvigson et al. reported acceptable doses to the small bowel, bladder, and femoral heads following LPLN boost; whereas Nayak et al. highlighted the need for longer follow-up beyond the median 32 months to evaluate potential late toxicities, including cystitis and bowel perforation, depending on the proximity of the boosted node to surrounding organs at risk [24, 25].

In addition to direct treatment-related toxicity, the potential impact of nodal RT boost on post-operative outcomes was evaluated by Li et al. [18], who reported no statistically significant difference in overall complication rates between the boosted and non-boosted groups (21.8% vs. 12.2%, *p* = 0.198), although a numerically higher rate of complications was observed in the boost cohort.

## Discussion

The findings of this review suggest that radiation boost to radiologically involved LPLNs in LARC is technically feasible and appears to have an acceptable safety profile in selected patients. Across the included retrospective studies, reported toxicity rates were generally low, and improvements in local control and reductions in lateral pelvic recurrence were observed; however, these findings should be interpreted cautiously given heterogeneity in patient selection, treatment approaches, and outcome definitions. Importantly, this strategy aligns with the broader shift toward organ-preserving and less invasive management approaches in rectal cancer [29]. Current evidence suggests that selective dose escalation to involved LPLNs may represent a promising strategy warranting further investigation, but its clinical benefit remains to be established in prospective studies.

Although several studies demonstrate benefit of RT boost in achieving nodal response, reported rates vary from 62.3% to 100%. This variation likely reflects differences in how nodal involvement was defined radiologically, rather than differences in treatment efficacy. The study reporting the lower response rate included nodes ≥ 5 mm with suspicious features, potentially capturing a broader group with lower rates of actual malignant involvement [25]. By contrast, the higher response rate was seen in a study that used a more selective threshold of ≥ 7 mm, aligning more closely with current guideline recommendations [21]. This supports the idea that stricter, standardised imaging criteria could improve comparability across studies and more accurately reflect treatment effect.

The comparative studies included in this review should be interpreted in the context of substantial potential selection bias and residual confounding. Patients selected for LPLN boost may differ systematically from control cohorts with respect to baseline nodal characteristics, extent of nodal disease, response to neoadjuvant therapy, chemotherapy regimens, surgical management strategies, institutional treatment protocols, and other prognostic factors. Importantly, comparator cohorts were not always equivalent to the boost groups in terms of baseline disease characteristics. For example, in the studies by Chen et al. and Hassanzadeh et al., patients in the non-boosted cohorts did not have radiologically involved LPLNs, whereas the boost cohorts specifically included patients with suspicious or involved LPLNs [22, 23]. In addition, variation in neoadjuvant treatment strategies was observed across some studies, including differences in the use of neoadjuvant chemotherapy versus neoadjuvant chemoradiotherapy [19]. These factors may independently influence tumour response and oncologic outcomes, making it difficult to attribute observed differences solely to radiation boost.

Furthermore, the absence of a clear and standardised definition of radiologically suspicious nodes introduces ambiguity not only in study reporting but also in clinical decision-making regarding LPLND. In such settings, RT boost may serve as a more practical and less invasive alternative, particularly in centres without the surgical expertise for LPLND. On a case-by-case basis, this strategy could be preferable in mitigating the morbidity associated with dissection.

When comparative analyses were performed, the retrospective nature of these studies often meant the lack of a standardised treatment protocol or uniform baseline characteristics. For example, in Li et al. [18], although SIB-IMRT was associated with a significantly lower 2-year local recurrence rate (0% vs. 10.8%, *p* = 0.024), this cohort had a significantly higher proportion of patients receiving concurrent CapOx chemotherapy. The same study also reported a higher rate of post-operative complications in the boosted group, although this was not statistically significant. This observation introduces an important clinical consideration. Pelvic surgery following radiotherapy is often more technically demanding due to radiation-induced fibrosis, tissue adhesions, and loss of normal anatomical planes [30, 31]. These changes may be further amplified when higher radiation doses are delivered to the lateral pelvic compartment. Theoretically, in cases requiring salvage LPLND or pelvic exenteration, particularly involving the lateral pelvic sidewall, RT boosting may increase operative difficulty and perioperative morbidity. Evidence from non-exenterative pelvic surgery following radiotherapy suggests increased technical complexity and complication rates [31]. However, there is currently no direct evidence in the literature to confirm or refute this concern in the context of exenterative surgery, underscoring another area where further study is needed.

Across studies, simultaneous integrated boost (SIB) was the predominant dose-escalation technique, with only one study also reporting the use of a sequential boost approach. The predominance of SIB is consistent with contemporary radiotherapy practice, in which SIB is widely used to facilitate dose escalation without prolonging the overall treatment course. However, no studies directly compared SIB with sequential boost for LPLN irradiation, and the available evidence is therefore insufficient to determine whether one technique offers superior oncological or toxicity outcomes.

Reporting of image-guidance techniques was limited across the included studies. Büttner et al. utilised daily cone-beam CT (CBCT)-based image guidance for treatment delivery, while Hassanzadeh et al. and Geng et al. used MRI fused with planning CT for target delineation [20, 23, 27]. However, most studies did not describe image-guidance protocols in sufficient detail, limiting assessment of the reproducibility and precision of LPLN boost delivery.

This review has several limitations. First, the search strategy was restricted to English-language publications, which may have introduced language bias and resulted in the omission of potentially relevant studies published in other languages. Additionally, publication bias cannot be excluded, as studies reporting favorable outcomes may be more likely to be published than studies with negative or inconclusive findings.

There is considerable heterogeneity across studies with respect to boost dose, delivery technique, imaging criteria for nodal involvement, outcome definitions, and concurrent chemotherapy regimens. The wide variation in both total dose and method of boosting substantially limits the generalisability of the available evidence. Furthermore, the inconsistent use of expansion margins from the GTVn highlights the absence of a standardised approach and underscores the need for consensus guidelines to inform target volume delineation in LPLN boosts. These methodological differences limit the generalisability of our findings and posed challenges in pooling data, particularly for nodal response, overall survival, disease-free survival, and toxicity outcomes. A clear need exists for consensus guidelines outlining the indications for RT boost, including standardised imaging criteria for involvement, dose parameters, and consistent reporting of outcomes such as nodal response, local control, and treatment-related toxicity. 

Although several studies reported toxicity outcomes and provided information regarding dose exposure to organs at risk (OARs), toxicity outcomes specifically related to LPLN dose escalation, including vascular injury, lymphoedema, and other late pelvic complications, were not consistently assessed or reported. The absence of standardised reporting of OAR constraints and LPLN-specific late toxicity outcomes limits assessment of the long-term safety and reproducibility of this approach. Future prospective studies should incorporate detailed reporting of radiotherapy planning parameters, OAR constraints, and long-term toxicity outcomes to better define the safety profile of LPLN boost strategies.

## Conclusion

Despite current limitations in the literature, radiotherapy boost to involved LPLNs remains a promising strategy that warrants further prospective evaluation to define its role in optimizing outcomes for patients with rectal cancer. Future research should focus on well-designed prospective trials that directly compare RT boost with LPLND in patients with radiologically suspicious LPLNs, to determine whether boost can offer equivalent oncological control with fewer functional consequences. Future trials should also assess functional outcomes, particularly urinary and sexual morbidity, and explore imaging-based biomarkers that predict nodal radioresistance. Integration of these findings into international guidelines will be essential to harmonise global practice. In addition, the development of consensus guidelines is essential to standardise imaging criteria for nodal involvement, target volume delineation, dose constraints, and outcome reporting. These efforts will be crucial in refining patient selection and integrating RT boost to LPLNs into evidence-based multidisciplinary pathways for rectal cancer.

## Electronic Supplementary Material

Below is the link to the electronic supplementary material.

Supplementary Table 1. Search strategy for systematic review. (DOCX 15.1 KB)

## Data Availability

No datasets were generated or analysed during the current study.
