# Supplementary material for: Evidence Gap in Managing Lateral Pelvic Lymph Nodes in Rectal Cancer: a Systematic Review of Radiation Boost Strategies
Source: J Gastrointest Cancer. 2026 Sep 7;57(1):212. doi: 10.1007/s12029-026-01580-1 (PMC13550032; doi:10.1007/s12029-026-01580-1)
Supplement: Supplementary file 1 [file 12029_2026_1580_MOESM1_ESM.docx]

| **Medline** | **Embase** | **Clinicaltrials.gov** | **Cochrane database** |
| --- | --- | --- | --- |
| ((rectal cancer or rectal neoplasm*) and (lateral pelvic lymph node* or pelvic lymph node* or pelvic sidewall or nodal irradiation or pelvic radiotherapy or extramesorectal lymph node* or obturator node*) and (radiotherapy or radiation therapy or intensity modulated radiotherapy or IMRT or volumetric modulated arc therapy or VMAT or simultaneous integrated boost or SIB or external beam radiation therapy or radiation boost or dose escalation)).mp. | ((rectal cancer or rectal neoplasm*) and (lateral pelvic lymph node* or pelvic lymph node* or pelvic sidewall or nodal irradiation or pelvic radiotherapy or extramesorectal lymph node* or obturator node*) and (radiotherapy or radiation therapy or intensity modulated radiotherapy or IMRT or volumetric modulated arc therapy or VMAT or simultaneous integrated boost or SIB or external beam radiation therapy or radiation boost or dose escalation)).mp. | Rectal cancer and simultaneous integrated boost;  Rectal cancer and intensity modulated radiotherapy | Rectal cancer and simultaneous integrated boost;  Rectal cancer and intensity modulated radiotherapy |

Supplementary Table 1. Search strategy for the systematic review.
